# New approaches to selecting a scan-sampling method for chicken behavioral observations and their practical implications

**DOI:** 10.1038/s41598-023-44126-2

**Published:** 2023-10-11

**Authors:** Alice Cartoni Mancinelli, Angela Trocino, Laura Menchetti, Diletta Chiattelli, Claudia Ciarelli, Cesare Castellini

**Affiliations:** 1 Department of Agricultural, Food and Environmental Sciences, University of Perugia, 06100 Perugia, Italy; 2 Department of Agronomy Food Natural Resources Animal and Environment (DAFNAE), University of Padova, Viale dell’Università 16, 35020 Legnaro, Padua Italy; 3 School of Biosciences and Veterinary Medicine, University of Camerino, Via della Circonvallazione 93/95, 62024 Matelica, Macerata Italy

**Keywords:** Behavioural ecology, Biodiversity, Animal behaviour

## Abstract

The use of the scan-sampling method, especially when a large amount of data is collected, has become widespread in behavioral studies. However, there are no specific guidelines regarding the choice of the sampling interval in different conditions. Thus, establishing a standard approach for video analysis represents an important step forward within the scientific community. In the present work, we hypothesized that the length of the sampling interval could influence the results of chicken behavioral study, for which we evaluated the reliability, accuracy, and validity of three different sampling intervals (10, 15 and 30 min). The Bland–Altman test was proposed as an innovative approach to compare sampling intervals and support researcher choices. Moreover, these sampling intervals were applied to compare the behavior of 4 chicken genotypes kept under free-range conditions. The Bland–Altman plots suggested that sampling intervals greater than 10 min lead to biases in the estimation of rare behaviors, such as “Attacking”. In contrast, the 30-min sampling interval was able to detect differences among genotypes in high-occurrence behaviors, such as those associated with locomotory activity. Thus, from a practical viewpoint, when a broad characterization of chicken genotypes is required, the 30-min scan-sampling interval might be suggested as a good compromise between resources and results.

## Introduction

The interconnections of animal, human and environmental welfare play a pivotal role in production chains. Such interconnection has particular relevance in the present, as Europe redesigns the agricultural and livestock system, by defining the main pillars, with the ambitious goal of becoming the first climate-neutral continent by 2050. From this perspective, the livestock sector is a candidate participant in the legislative process regarding the European Farm to Fork and the European Green Deal strategies^1^. This necessary and complex challenge towards sustainable and alternative production is driving research to deeper issues such as animal welfare and low-input rearing systems. In particular, in alternative rearing systems (organic and free-range) characterized by pasture presence, animals exhibit a larger ethogram than those reared in conventional systems^2,3^. Under this framework, experimental designs and settings involving behavioral observations are increasingly complex, as applied ethology aims to evaluate animal responses to specific rearing systems (e.g., conventional or alternative) or conditions (e.g., heat stress, animal density, dietary strategies)^3–6^. Among the hot topics in sustainable poultry systems, an open issue is the choice of genotypes for alternative production (outdoor and/or organic) and their ability to adapt to an outdoor environment. In this context, studies often need to include many animals from various genotypes, long observation periods (as the rearing cycle of poultry has a minimum duration of 81 days), and daily repetitions (as poultry exhibit a circadian rhythm) besides the recording of positive behaviors, such as foraging- and comfort-related behaviors, and negative behaviors, such as stereotypies^7^.

The use of technological devices such as cameras, sensors, and motion detectors, along with the introduction of computational animal behavior analysis (CABA), make behavior data acquisition easier and eliminate the bias caused by the presence of the observer^8^. The widespread use of indirect observations and smart technologies has increased the reproducibility of animal behavior studies^9,10^. However, the field has yet to establish a common methodology for video analysis, which remains an open issue. Most of the recently published papers^11–14^ have adopted the scan-sampling method for video analysis. In the scan-sampling method, observations are divided into sampling intervals, and the animal’s behavior is recorded periodically at the end of each sampling interval, i.e., sampling point^15–17^. However, the ideal sampling interval, or the time periods into which the observation is divided, is largely debated. The interval length is usually determined according to the research question, characteristics of the observed animals, and their activity level. Moreover, the total duration of the observation period and the number of animals involved could guide the decision because it influences the number of scans and the time required to analyze each scan. As a rule of thumb, the more active the animal is, the more variable its behavior; thus, shorter intervals should be selected. Conversely, a longer interval could be used to observe inactive animals. However, animal activity can be influenced by several variables, the first of which is the environmental context in which animals are kept, and there are no specific guidelines. Thus, the choice of sampling intervals for analyzing poultry behavior remains unclear, with intervals in recent studies ranging from 2 to 60 min^14,18^. The reliability and the validity of the results of such varying sampling intervals (i.e., the extent to which they can be reproduced and the extent to which really measure what they are supposed to measure, respectively^17^) have not yet been investigated although it would be crucial to ensure experiment reproducibility and trustworthiness. In addition to these questions, an important practical consideration is the feasibility (i.e., the extent to which the proposed measurement procedure is possible, practicable and worthwhile^17^). A compromise between accuracy and feasibility is always necessary, taking into account the aim of the study.

Thus, in the present study, we hypothesized that the length of the sampling interval could affect the results and, consequently, the conclusions drawn and the interpretations made about poultry behavior. Therefore, we evaluated the reliability, accuracy, and validity of three different sampling intervals (i.e., 10, 15, and 30 min) for the behavioral observations of broiler chickens. The reliability of each interval was investigated by agreement analyses. Then, sampling intervals were compared using an innovative approach for ethological studies (i.e., Bland–Altman analysis), and biases were critically analyzed to determine the appropriate recording rules for behavioral observations in poultry considering feasibility and accuracy. Finally, the three sampling intervals were applied in a real experimental context to analyze their validity. In particular, the effect of chicken breed (four breeds, i.e., Red, LD, NN, and CB) on the behavior observed using the three sampling methods was quantified to investigate whether (i) the length of the interval influenced the statistical inferences and (ii) intervals over 10 min are valid for comparing outdoor behavior among genotypes characterized by very different attitudes.

## Results

### Time required and feasibility of analysis

**Figure 1:**
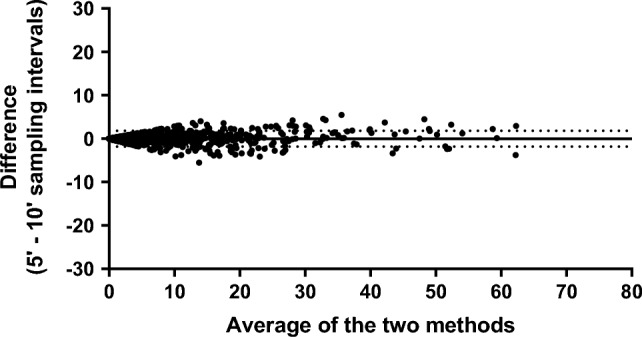
Bland–Altman plots of differences (as raw values) between data collected with 5- and 10-min sampling intervals in the mean proportion of animals engaging in each behavior per scan. The solid line represents the bias (− 0.0003), while the dashed lines represent the limits of agreement (− 1.836 to 1.835).

Data collection required approximately 7 min per scan. Intervals shorter than 10 min, involving many scans/session, are therefore very time-consuming. In the preliminary analyses, we calculated the occurrences using a sampling interval of 5 min (i.e., 24 scans/session (1 session = 2 h); Supplementary Table S1). This implied over 2.5 h of work for each session. The occurrences recorded with this method, moreover, showed an almost perfect agreement with those collected at 10 min (all Intraclass Correlation Coefficients (ICCs) > 0.9; Supplementary Table S2) and a bias that we considered negligible (bias = − 0.0003; Limits of Agreement (LoA) =  − 1.836 to 1.835; Fig. 1). In light of these results and the feasibility of the analyses, we have considered the 10-min interval as "the shortest applicable" under the conditions of the present study.

### Comparison with the continuous method and definition of the best estimate of the true values

The 10-min interval showed the highest R^2^ as well as the lowest Mean Absolute Error (MAE) and Root Mean Square Error (RMSE) in the comparisons with the continuous sampling method (Table 1). It was therefore the best estimate of the true occurrences and was considered as the reference sampling interval in subsequent analyses.

**Table 1:** Parameters used to choose the scan-sampling method that provided the best estimate of true occurrences (i.e., values obtained by continuous sampling method).

Scan-sampling intervals	R^2^	MEA	RMSE
10 min	0.521	3.73	7.68
15 min	0.435	3.93	8.33
30 min	0.386	4.58	8.69

R, R-Square of the regression model including the continuous sampling method as the dependent variable; MAE, Mean Absolute Error; RMSE, Root Mean Square Error.

### Reliability of the different sampling intervals

The Intraclass Correlation Coefficients** (**ICC) indicated good (0.60 ≤ ICC < 0.75) or excellent (ICC ≥ 0.75) interobserver agreement for all the recorded behaviors. The lowest ICC values (< 0.75) were obtained for Allo-grooming in the 15- and 30-min sampling intervals; Running had perfect agreement (ICC = 1.000) for all sampling methods (Supplementary Tables S3).

ICC values were also used to evaluate the agreement among the 3 methods (i.e., 10-, 15-, and 30-min sampling intervals) for each behavior (Table 2). The ICC values indicated good or excellent agreement among the rates of animals observed engaging in the different behaviors yielded by the 3 intervals. Eleven out of 19 behavioral recordings had ICC values > 0.9, while 4 recordings had ICC values < 0.8; this indicates that the three sampling intervals yielded highly consistent results for most of the behavioral variables. The variables with the lowest ICC (i.e., Feed pecking, Attacking, Escaping, and Allo-grooming) were all low frequency behaviors, suggesting that the greatest differences among sampling intervals occurred in rare behaviors.

**Table 2:** Agreement among the 3 methods. Intraclass correlation coefficients (ICCs) of the behavioral variables assessed by Observer A using the three sampling intervals (i.e., 10, 15, and 30 min sampling intervals). Each ICC is followed by its 95% confidence interval (CI) and by the *p* value of the F test.

Behavior	ICC	95% CI (lower bound)	95% CI (upper bound)	*p* value
High-occurrence behaviors				
Roosting	0.970	0.954	0.981	***
Walking	0.939	0.908	0.961	***
Grass pecking	0.918	0.876	0.947	***
Resting	0.906	0.858	0.940	***
Other pecking	0.955	0.931	0.971	***
Self-grooming	0.916	0.872	0.946	***
Medium-occurrence behaviors				
Hiding	0.970	0.955	0.981	***
Running	0.905	0.856	0.939	***
Dust bathing	0.979	0.968	0.986	***
Wing flapping	0.902	0.851	0.937	***
Drinking	0.865	0.795	0.913	***
Scratching	0.924	0.884	0.951	***
Low-occurrence behaviors				
Stretching	0.840	0.757	0.897	***
Attacking	0.776	0.661	0.857	***
Feed pecking	0.765	0.645	0.850	***
Escaping	0.744	0.612	0.836	***
Allo-grooming	0.770	0.652	0.853	***
Swelling	0.873	0.808	0.919	***
Sleeping	–	–	–	–

***p < 0.001; –not calculated due to zero variance.

### Differences among sampling methods

**Figure 2:**
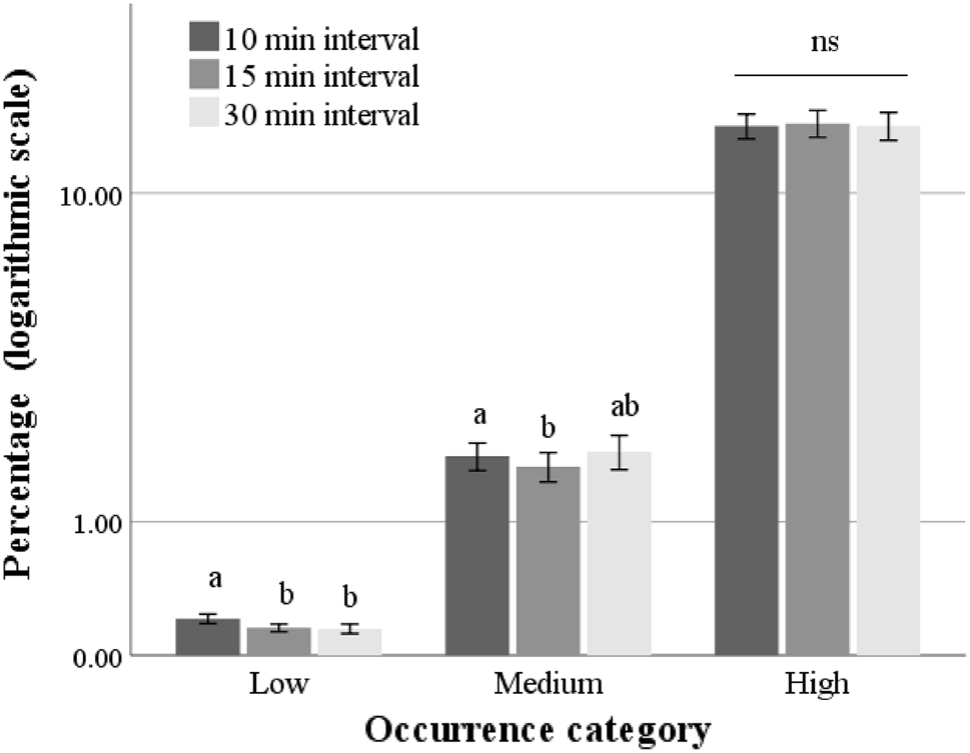
Percent of animals performing the different behaviors (% of all visible animals ± standard errors) per scan under the different sampling intervals. The behaviors are categorized according to their frequency of occurrence (low, medium, and high). A logarithmic scale was used for the y-axis to facilitate the visualization of data with very different frequencies.

Figure 2 and Table 3 show the descriptive statistics of behavioral recordings and comparisons among the occurrences of each behavior obtained by the three sampling methods (i.e., 10-, 15-, and 30-min sampling intervals). The 10-min sampling interval yielded the highest occurrence of rare behaviors (*p* < 0.001) and, in particular, aggressive behaviors (i.e., Attacking; *p* = 0.011; Table 3). The 10-min sampling interval also yielded higher occurrences of behaviors in the medium-occurrence category (*p* = 0.035) compared to the 15-min sampling interval. These findings suggest that a 10-min sampling interval provides the best accuracy for these behavioral categories. The differences were not significant for high-occurrence behaviors (*p* = 0.552), although the analysis of individual variables revealed differences among sampling intervals in the values obtained for Resting (*p* = 0.005) and Roosting (*p* = 0.022; Table 3).

**Table 3:** Animals performing the different behaviors (% of all visible animals ± standard deviation) under the three sampling methods (i.e., 10-, 15-, and 30-min sampling intervals).

Behavior	Sampling interval			*p* value
	10-min interval	15-min interval	30-min interval	
High-occurrence behaviors				
Roosting	31.34^b^ ± 7.99	33.35^a^ ± 8.82	32.02^ab^ ± 10.89	0.022
Walking	17.61^a^ ± 3.13	18.07^a^ ± 5.11	19.29^a^ ± 6.02	0.050
Grass pecking	14.39 ± 2.72	15.05 ± 3.31	14.85 ± 3.49	0.269
Resting	13.49^a^ ± 5.13	12.16^ab^ ± 4.66	10.98^b^ ± 5.37	0.005
Other pecking	6.29 ± 2.31	5.89 ± 1.85	6.63 ± 2.53	0.105
Self-grooming	4.33 ± 2.37	4.26 ± 2.91	3.77 ± 3.08	0.068
Medium-occurrence behaviors				
Hiding	3.50 ± 2.27	3.33 ± 2.41	3.45 ± 2.64	0.611
Running	2.78 ± 2.51	3.10 ± 2.58	3.76 ± 4.06	0.308
Dust bathing	1.49 ± 2.05	1.34 ± 1.81	1.46 ± 1.89	0.533
Wing flapping	1.43 ± 1.14	1.15 ± 0.69	1.24 ± 1.09	0.314
Drinking	0.92^a^ ± 0.75	0.43^b^ ± 0.48	0.58^ab^ ± 0.79	0.010
Scratching	0.75 ± 0.64	0.64 ± 0.54	0.78 ± 0.80	0.533
Low-occurrence behaviors				
Stretching	0.49 ± 0.45	0.41 ± 0.44	0.53 ± 0.60	0.098
Attacking	0.46^a^ ± 0.31	0.32^ab^ ± 0.25	0.24^b^ ± 0.31	0.011
Feed pecking	0.15^a^ ± 0.33	0.02^a^ ± 0.07	0.03^a^ ± 0.13	0.023
Escaping	0.13 ± 0.10	0.07 ± 0.11	0.10 ± 0.18	0.059
Allo-grooming	0.06 ± 0.08	0.02 ± 0.05	0.04 ± 0.10	0.163
Swelling	0.05 ± 0.09	0.05 ± 0.14	0.07 ± 0.26	0.229
Sleeping	0.00 ± 0.02	0.00 ± 0.00	0.00 ± 0.00	0.368
Others	0.32 ± 0.47	0.34 ± 0.21	0.18 ± 0.24	0.076

Values followed by the same letter in each row do not differ significantly (*p* < 0.05, Bonferroni corrected).

Bars not sharing any superscript within each category are significantly different at *p* < 0.05 (Bonferroni corrected).

**Figure 3:**
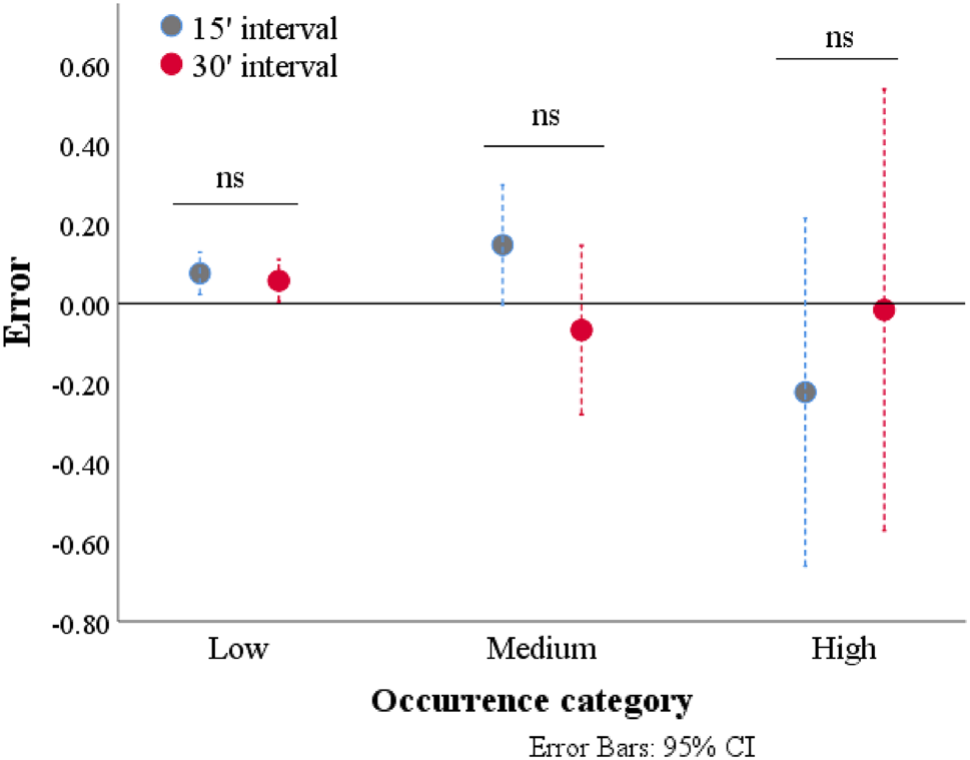
Mean error rates (with error bars displaying 95% confidence intervals (CI)) for the behavioral recordings with 15- and 30-min sampling intervals. Data from the 10-min sampling interval were treated as the reference values. ns = no significant difference between 15- and 30-min sampling intervals.

Figure 3 shows the mean error rates (and their 95% CIs) for the 15- and 30-min sampling intervals according to the category of occurrence. The 10-min sampling interval was treated as the reference value. The 95% CIs of the low-occurrence category did not contain the value 0, confirming that both 15- and 30-min intervals resulted in significant underestimation of these behaviors. An underestimation of behaviors included in the medium-occurrence category was also observed for the 15-min sampling interval. The CIs of the three behavioral categories also showed that the variability in the mean error rate increased as the magnitude of the occurrence increased. Finally, for all behavioral categories, no difference was found between the errors of the 15- and 30-min sampling intervals, suggesting that the use of the 15- and 30-min sampling intervals leads to similar errors.

**Figure 4:**
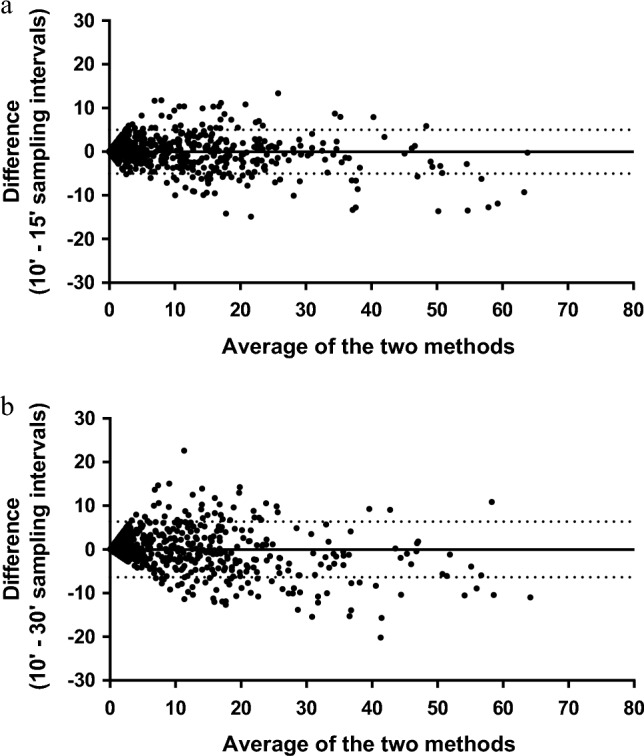
Bland–Altman plots of differences (as raw values) between data collected with 10- and 15-min sampling intervals (Panel **a**) and data collected with 10-min and 30-min sampling intervals (Panel **b**) in the mean proportion of animals engaging in each behavior per scan. The solid line represents the biases, while the dashed lines represent the limits of agreement.

Figure 4 shows the Bland–Altman plots, where the y-axis shows the difference, as raw values, between the occurrences yielded by the 10- and 15-min sampling intervals (Panel 4a) and those yielded by the 10- and 30-min sampling intervals (Panel 4b). The x-axis represents the average of the two methods, while the solid line and the two dotted lines indicate the bias and its Limits of Agreement (LoA), respectively. The biases were close to 0 (− 0.0003 ± 2.558% and − 0.0005 ± 3.265% for the 15- and 30-min intervals, respectively), although a progressive increase in the variability of the differences was found. Several biases lay outside the LoA (− 5.014 + 5.013% and − 6.399 + 6.398% for the 15- and 30-min intervals, respectively), reaching, in some cases, values higher than $\left| {10} \right|$.

**Figure 5:**
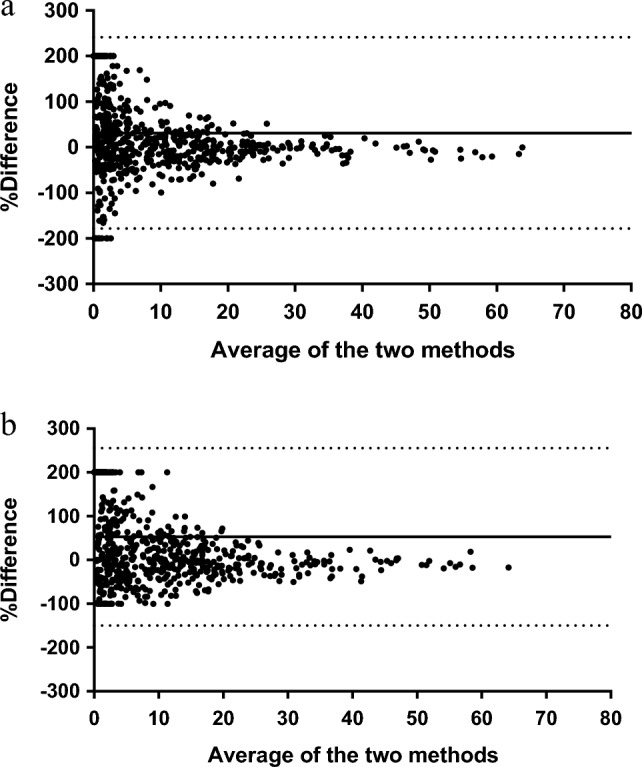
Bland–Altman plots of the percent differences between 10- and 15-min (Panel **a**) and between 10- and 30-min (Panel **b**) intervals. The solid line represents the bias, while the dashed lines represent the limits of agreement.

To understand the practical impact of biases on the estimation of occurrences, the most appropriate approach would be to evaluate them as a percentage of the total occurrence rather than as raw values. Figure 5 shows Bland–Altman plots where the differences between 10- and 15-min (Panel 5a) or between 10- and 30-min intervals (Panel 5b) are expressed as percentages of the expected values.

The differences were positive (31.47% and 52.84% for the 15- and 30-min intervals, respectively), and the LoA were wide ([− 178.3%, + 241.2%] and [− 149.9%, + 255.5%] for 15- and 30-min intervals, respectively), indicating that many values yielded by the 15- and 30-min intervals underestimate the percentage of animals performing that behavior and that there is substantial variability in the differences among the methods. The funnel-shaped arrangement of the points indicates that differences in the relative percentages tend to decrease as the occurrence of the behavior increases. Thus, the percentage of animals engaging in rare behaviors (dots on the left side of the graph) could be overestimated or underestimated by up to twofold compared to that under the 10-min sampling interval, while the relative differences are smaller for more frequent behaviors (dots on the right side of the graph). Therefore, the impact of long sampling intervals was severe for rare behaviors and negligible for more frequent behaviors.

### Differences among genotypes obtained using the three sampling methods

**Figure 6:**
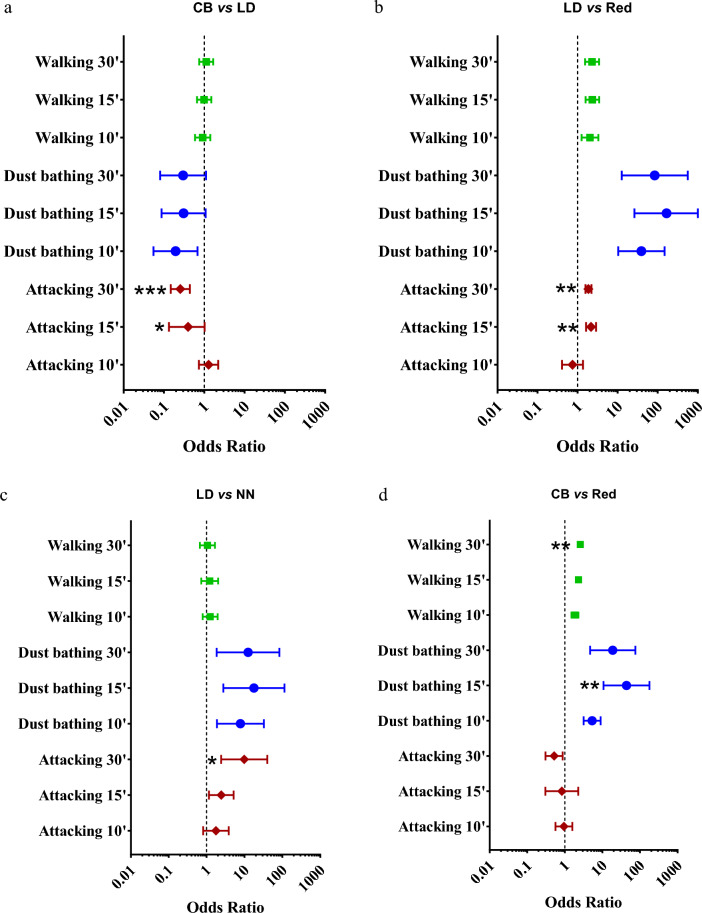
Odds ratios for the genotype effect (**a**, **b**, **c** and **d**) under the three sampling methods on high-, medium- and low-occurrence behaviors (Walking, Dust bathing and Attacking, respectively). ***p < 0.001, **p < 0.01, and *p < 0.05 versus 10-min interval data on each behavior.

The Odds Ratios (ORs) for the genotype effect were compared to evaluate whether the estimated association between behavior and genotype was influenced by the sampling method (Figs. 6 and Supplementary Figure S1). For three out of six comparisons between genotypes (i.e., CB vs. LD, LD vs. Red, and LD vs. NN; Fig. 6a–c), there was a difference in the OR of Attacking estimated by the three sampling methods (*p* < 0.05). These differences provided different evidence against the null hypothesis of OR (i.e., OR = 1) and thus about the association between genotype and behavior. Specifically, there was no difference in Attacking between CB and LD chickens sampled every 10 min (OR = 1.277, 95% CI = 0.736–2.214; *p* = 0.384), while Attacking was lower in CB than in LD chickens when sampled every 15 and 30 min (*p* < 0.05; Fig. 6a). Differences among the ORs estimated by different sampling intervals were also found for Walking and Dust bathing in the comparison between CB and Red chickens (*p* < 0.01; Fig. 6d). However, these differences did not influence the interpretation of the ORs, as Walking and Dust bathing were significantly higher in CB chickens than in Red chickens with all sampling methods.

## Discussion

Behavioral analyses are used for a large number of topics ranging from ethology to more applicative studies, making them invaluable in the assessment of animal welfare and adaptability. In this context, the use of the scan-sampling method has become widespread; although it entails a partial loss of data compared to the continuous method, it represents a useful approach when a large amount of data is collected. Continuous behavioral sampling is still the gold standard for behavioral evaluations, as true frequencies and durations, the times at which behaviors stop and start, and the sequences of behaviors can be obtained for the entire duration of the video^17,19^. Nevertheless, Martin and Bateson^17^ state that “trying to record everything can mean that nothing is measured reliably”. The scan-sampling method is less demanding than the continuous method and facilitates the observation of multiple animals in several categories for a long period of time^17^, thus providing a compromise between accuracy and time savings. In particular, in studies similar to ours, which are characterized by a numerous of video recordings and a high number of animals, the use of continuous sampling is not feasible; thus, scan sampling appears to be the only possible option. However, the interval length for scan sampling is a complex choice that can affect the final results. Researchers often have no reference points for a reasoned choice and must solely rely upon their own experience. Researchers should, however, take into account the aim of the study, the characteristics of the animal, and the different factors that can influence the behavior of interest, such as the experimental setting and environmental context. One of the main goals of this study was to provide a reliable methodology to broadly compare different chicken genotypes without compromising the end results and considering the time spent by the observer for video analysis.

Thus, this study explored the behavioral analysis of chickens with a rigorous statistical approach that, in addition to the feasibility, investigated (i) the reliability (i.e., the agreements among observers and among sampling methods), (ii) the accuracy (i.e., error rate and biases), and (iii) the validity (correctness of the inferences in a practical application) of different sampling intervals^20^. First, the 10-min interval was considered "the shortest applicable" in our context based on preliminary evaluations that mainly took into consideration the feasibility (i.e., the time required for each scan and number of scans/session) and which excluded a sampling interval of 5 min. The 10-min interval was also chosen as a reference since, in comparisons with the continuous method, it demonstrated the best predictive ability of real occurrences and the smallest absolute error.

Afterward, the behaviors reported in the chicken ethogram were categorized into three main groups according to the frequency of the behavior (i.e., low-, medium-, and high-occurrence), as it is known that frequency can affect the reliability of recordings^20^. The obtained classification is consistent with other studies^21,22^ where it has been reported that broiler chickens spent 80% of their budget time in locomotor, stationary, and foraging behaviors, as verified by the high occurrence of Grass pecking, Other pecking, Walking, Resting, Roosting, and Self-grooming.

The frequency of behaviors influenced the interobserver agreement, as the lowest indices were obtained for the low-occurrence behaviors, such as Allo-grooming. As expected, categories of occurrence were also relevant for the agreement among sampling methods. In particular, the shortest sampling interval (10 min) had better performance in identifying low- and medium-occurrence behaviors compared to the 15- and 30-min intervals, whereas no differences among sampling intervals were found for the most frequent behavior. Comparison with previous studies is not easy since, despite the use of the same software for video analysis, rearing conditions of animals may be substantially different. In fact, when investigating alternative observation methods for behavioral evaluation in young broiler chickens kept individually indoors, Ross et al.^23^ affirmed that scan-sampling methods with an interval length above 5 min were inaccurate because the average duration of each behavior was under 30 s. It is widely known that the husbandry system can affect the expression of various behaviors, both in terms of frequency and duration; indeed, some specific behaviors, such as social interactions, were not expressed in the Ross et al.^23^ setting. While Ross et al.^23^ suggested an average length of less than 30 s for each poultry behavior, our observation length for each scan was 10 s, independent of the three scan intervals adopted. In this way, due to the high number of animals (50 birds/pen), the observers had the best possible conditions to evaluate animal behavior. Moreover, the presence of specific tools (zoom, slow-motion, etc.) in the Observer XT software allowed us to analyze the individual behaviors in each scan as precisely as possible.

The present study indicated that accuracy was affected by the sampling method according to the frequency of occurrence. In particular, the error analysis confirmed that the 15- and 30-min sampling intervals underestimated the occurrence of rare behaviors. However, interestingly, there was no difference between the estimated errors of these two methods. This could suggest that 15- and 30-min sampling intervals are interchangeable and that 30 min of scan sampling does not reduce the accuracy of the data compared to the 15-min interval. On the other hand, half of the sample points in the 30-min interval overlapped with the sample points of the 15-min interval. This could explain the lack of difference between the errors that were obtained by 10- and 30-min sampling intervals.

The accuracy and differences among methods were further investigated using Bland–Altman plots. Bland–Altman analysis is a graphical approach usually used to validate clinical measurements and as an indicator of agreement^24,25^, as it can quantify and visualize the differences among the values recorded with different methods. In agreement studies, these plots are preferable to the correlation coefficient, which measures the strength of a linear relation between two variables. Regarding behavioral studies in animals, this method has recently been used to determine the agreement between data obtained from collar-based sensors and human observations in dairy cows^26^. In our study, Bland–Altman plots confirmed that both 15- and 30-min intervals underestimated the percentage of animals performing a specific behavior and indicated that the absolute bias increased with increasing occurrences of the same behaviors. This bias could exceed 10% for both the 15- and 30-min sampling intervals.

The Bland–Altman plots where the bias was expressed as a percentage of the expected values better displayed the practical impact of these biases. This result indicated that the highest relative differences were found for rare behaviors. Specifically, the 15- and 30-min intervals over- or underestimated the “true” value by up to 2 times. Such a bias could have important consequences for data interpretation. Relative differences, however, decreased and tended to be negligible in the case of high-occurrence behaviors such as Roosting, Walking, Grass pecking, and Resting. In this regard, the following specific considerations must be made for the behaviors observed in chickens kept outdoors: (i) frequent behaviors comprise approximately 80% of chickens’ budget time^21,22^, (ii) they represent the most investigated behaviors for characterizing and comparing genetic strains^27^, and they are used to evaluate the adaptability of chickens under different rearing conditions^28^. Accordingly, when the study requires the evaluation of frequent behaviors, the 30-min scan-sampling interval might be a good compromise between resources (funding and time) and results. In fact, it can be assumed that the true value of frequent behaviors did not differ among the tested sampling intervals. In contrast, studies of rare behaviors (such as the evaluation of behavioral sequences in relation to stress conditions^29^, the fear response, or positive behaviors, such as playing^30^) necessitate very short intervals or continuous recording.

These considerations were strongly supported by the application of the three sampling intervals to evaluate the behavioral differences among genotypes. Our data showed that for a rare behavior, such as Attacking, the scan interval influenced the OR, resulting in conflicting conclusions regarding the genotype behavior relationship. In particular, both the 15- and 30-min sampling intervals indicated a difference in Attacking between LD and CB chickens, which was not found by using the 10-min interval. Misinterpretations of this behavior were also found in the comparisons between LD and Red chickens and between LD and NN chickens. Quantitative differences could also emerge for the high- and medium-occurrence behaviors (e.g., Walking and Dust bathing), but in this case, they did not lead to different interpretations of the relationship between breed and behavior.

The results of this applied inferential statistic (i.e., the OR) confirmed the indications of the Bland–Altman plots and, in particular, the plots constructed using the bias expressed as a percentage of the expected values. Both approaches suggested that the 30-min scan-sampling interval provided a valid evaluation of high-occurrence behaviors useful for broadly characterizing chicken genotype, but the bias in rare behaviors such as Attacking could compromise the validity of these estimations. Thus, Bland–Altman analysis appears to be a useful tool to compare sampling intervals and inform researcher choices, as it provides an effective visual representation and allows practical considerations based on the practical significance of the bias.

The main limitation of this study is that the comparison among continuous and scan-sampling method was performed only for one session/genotype. However, this choice was amply motivated by the feasibility of assessing a large number of animals per pen and the many behavioral variables included in the ethogram.

## Conclusion

The present study highlights the importance of selecting a method for studying chicken behavior. It also demonstrates the need to define a reference sampling interval and proposes a multistep approach to reach a good compromise among the accuracy of the results, the aim of the research, and the available resources (e.g., funding and time). This approach also included a novel use of Bland–Altman analysis. The present findings indicated that a 30-min sampling interval could be applied in complex experimental designs characterized by a high number of chickens and the comparison of several experimental factors (e.g., genotypes, diets) if the aim is to characterize broad behavioral differences, such as locomotor activity and foraging. Conversely, sampling intervals shorter than 10 min are necessary for studies requiring the analysis of rare behaviors, such as aggression, stereotypies, and allopreening. Further studies could better define the length of these sampling intervals and the methods to be adopted under different breeding systems.

## Methods

This in vivo study was carried out from April to June 2020 in the experimental farm of the Department of Agricultural, Food and Environmental Sciences (University of Perugia, Italy).

**Figure 7:**
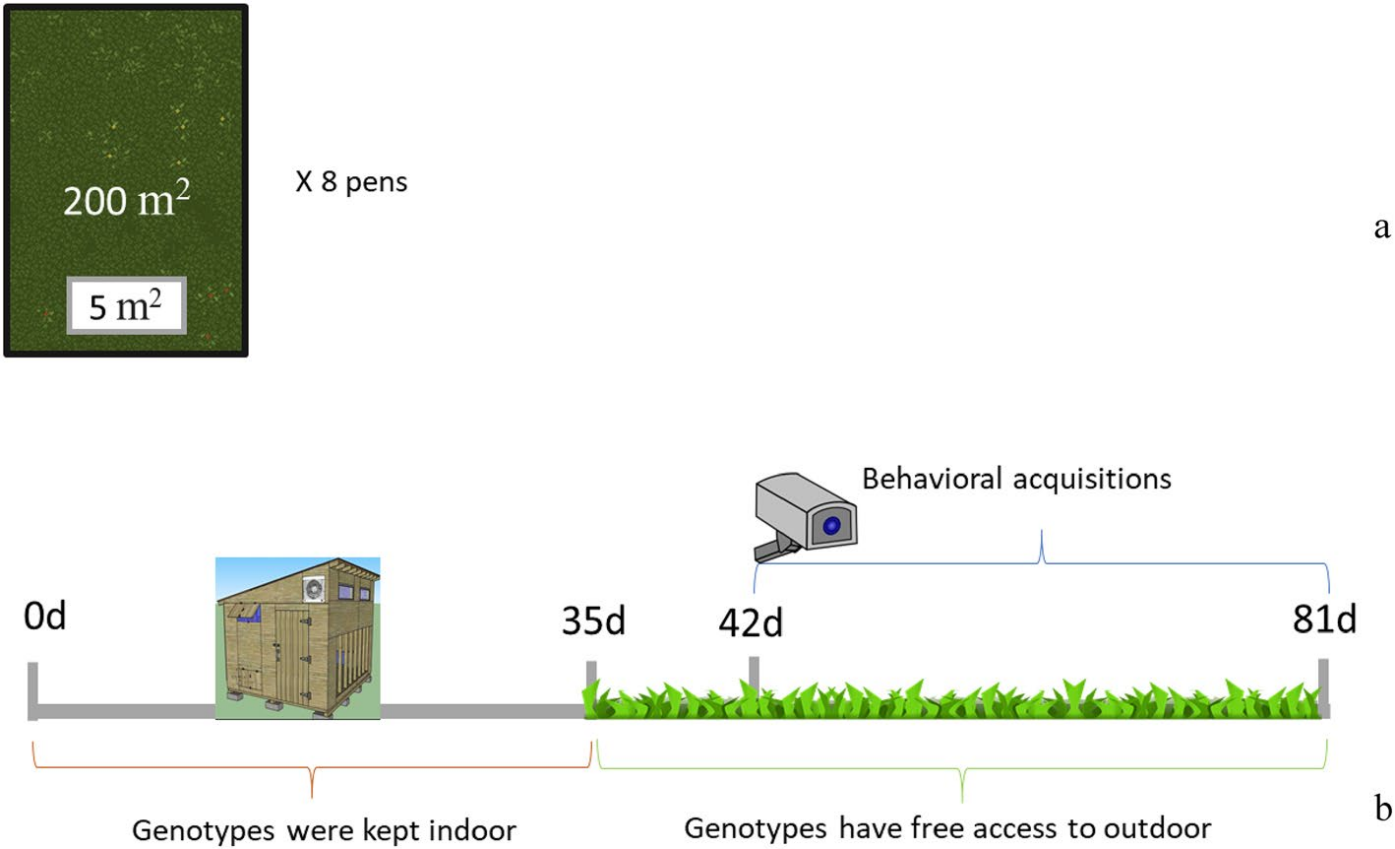
Experimental area (Panel **a**) and timeline (Panel **b**) of the trial.

The test area consisted of eight outdoor pens (200 m^2^/pen), each equipped with a shelter (5 m^2^), two feeders, and two drinkers (Fig. 7a).

Four different slow-growing (SG) chicken genotypes (Red, LD, NN, and CB) were used, and a total of 400 one-day-old chicks (100 chicks/breed of both sexes) were randomly housed in 8 pens with outdoor access (2 pens per breed; 50 animals each, 25 females and 25 males).

Until 35 days of age, animals were separately reared indoors and subjected to environmentally controlled parameters with a relative humidity between 65 and 70% and a temperature between 30 and 32 °C during the first week. Then, the temperature was decreased by 2 °C each week until it reached 24–26 °C. All chicks were vaccinated against coccidiosis, infectious bronchitis, Marek, Newcastle, and Gumboro and fed the same starter period diet. At 35 days of age, chickens were allowed access to the outdoor area during the day and kept in the shelter to protect them from predators during the night (0.10 m^2^/bird indoor density, 4 m^2^/bird outdoor density).

In May and June, animals had free access to the pasture; at the farm, the maximum average temperature was 24 °C and the minimum was 12 °C. The pasture was not treated with pesticides. During the trial, water and feed were always provided ad libitum, and all the genotypes were fed the same growth period diet.

The videos for the behavioral evaluations (see the Behavioral Observations section) were recorded from 42 days of age until the end of the rearing cycle (81 d). Figure 7b shows the trial timeline. The experimental protocol was approved by the Ethical Committee of the University of Perugia (ID: Prot.62705_2020). All methods were performed in accordance with the European legislation for the protection of chickens kept for meat production^31^, the protection of animals at the time of killing^32^, and the protection of animals used for scientific purposes^33^.

### Behavioral observations

Behavioral observations were conducted in all eight pens with the use of a computerized system (Noldus Technology, Wageningen, The Netherlands) consisting of two different software programs: Media Recorder, to record the videos with the use of eight cameras (BASLER, ac A 1300–60 gc), and Observer XT, to analyze the videos. As previously mentioned, the animals were free to explore the pasture starting from the fifth trial week (corresponding to 35 days of age), while video acquisition started in the sixth week (corresponding to 42 days of age). The period from the fifth to sixth weeks was considered a period of habituation and familiarization to allow animals to become confident in the outdoor area. During this period, the eight pens were inspected to establish both the camera position and the best time of day for recordings based on the highest expressed animal activity. In each outdoor pen, the camera was located 2 m above the ground such that the field of view was 20 × 10 m area/camera. Therefore, the camera covered all of the pen area of 200 m^2^.

From the sixth week to the end of the trial (81 d, Fig. 7b), 2 videos/week of 2 h length (9.00–11.00 AM) were recorded in each pen, for a total of 10 videos/breed (5 videos/replicate).

Two trained observers with extensive experience in poultry behavior (Observer A, Observer B) scored the videos to record the number of animals engaged in the different behaviors by the use of the reported ethogram (Table 4).

**Table 4:** Description of recorded behaviors of broiler chickens in the outdoor area (modified according to^6,34^).

	Behavior	ATOL references^1^	Description
Static	Roosting	ATOL_0000837	Chicken lying down with the underside in contact with the floor
	Resting	ATOL_0000816	Chicken standing with body parallel to the ground, head erect and eyes opened. Only the feet are in contact with the ground
	Sleeping	ATOL_0000873	Chicken sleeping with the head in a low position (under the wing or on the ground) and eyes closed
Active	Walking	ATOL_0000805	Chicken moving more than three steps
	Running	ATOL_0000806	Chicken quickly moving more than three steps
	Hiding	ATOL_0000814	Chicken moving through grass and bushes to find place to hide
Eat	Feed pecking	ATOL_0000363	Chicken pecking inside the feeder
	Drinking	ATOL_0000361	Chicken pecking inside the drinker
	Grass pecking	ATOL_0000844	Chicken pecking the grass
	Other pecking	ATOL_0000845	Chicken pecking other things
Comfort	Self-grooming	ATOL_0000823	Chicken preening its own feathers
	Scratching	ATOL_0000360	Chicken scratching the ground with its foot
	Stretching	ATOL_0000822	Chicken stretching its body and legs
	Wings flapping	ATOL_0000822	Chicken beating its wings with breast protruding and a vertically extended posture
	Swelling	ATOL_0005361	Chicken puffing out the breast feathers
	Dust bathing	ATOL_0000824	Chicken forcing sand or other material into its plumage by squatting on the ground and making appropriate movements with the body, wings and leg
Interaction	Attacking	ATOL_0000813	Chicken fighting with a conspecific
	Escaping		Chicken escaping from a conspecific
	Allo-grooming	ATOL_0000826	Chicken preening the feathers of a conspecific

^1^Traits follow the ATOL ontology (https://www.atol-ontology.com/en/atol-2/), in accordance with the PILLOW project data management plan.

**Figure 8:**
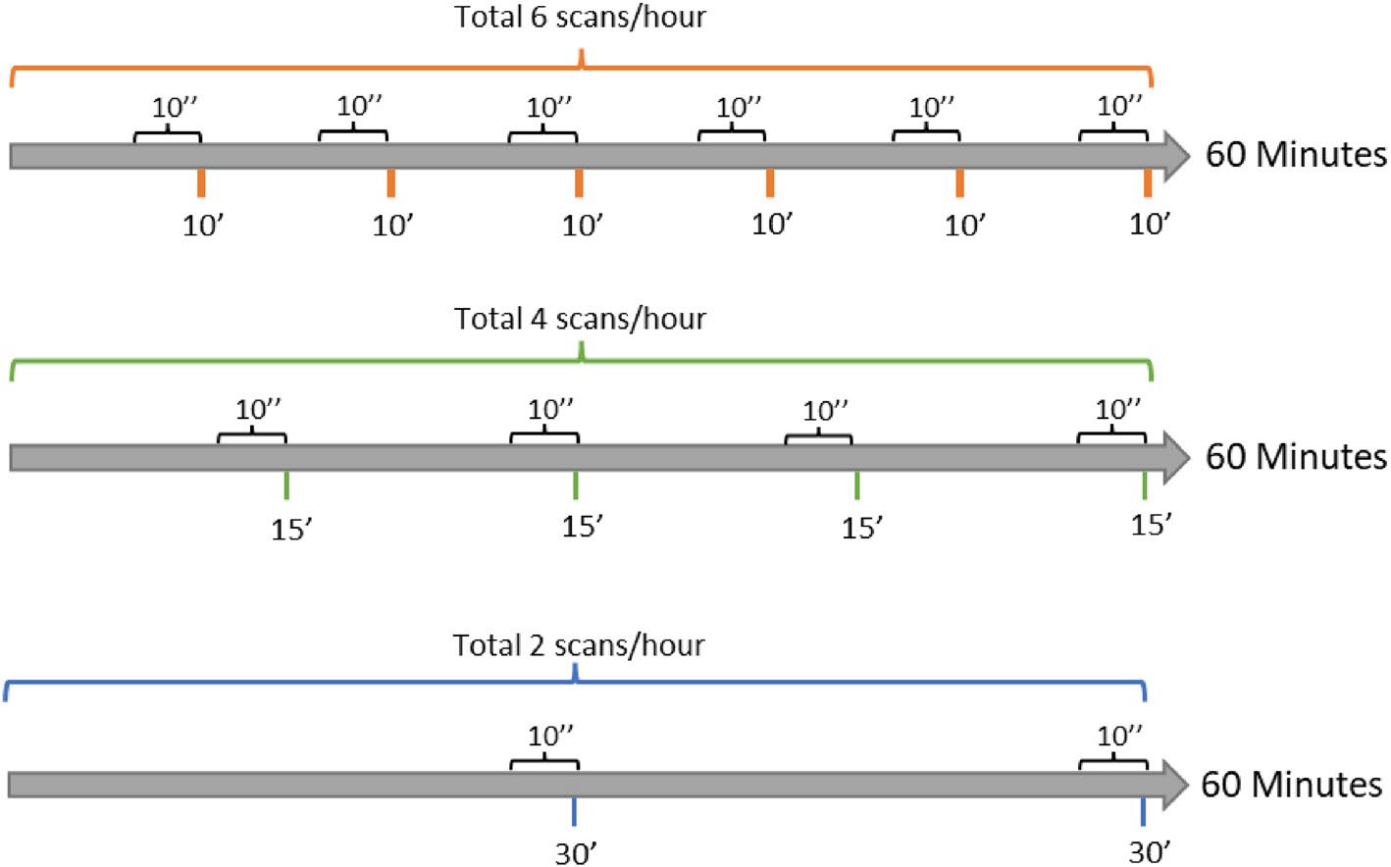
Graphical representation of the sampling intervals applied in the study (10, 15, and 30 min). Each scan was involved the same length of observation (10 s).

Each video was analyzed using three different sampling intervals (Fig. 8):

1. 10 min (6 scans per hour; 12 scans per video);
2. 15 min (4 scans per hour; 8 scans per video);
3. 30 min (2 scans per hour; 4 scans per video).

In the preliminary analyses, a 5-min interval (12 scans per hour; 24 scans per video) was also applied. Moreover, the continuous sampling method was used for a selection of videos (i.e., 4 sessions randomly chosen for each genotype (2 sessions/replicate)) in order to choose the sampling interval to use as a reference (see paragraph Statistical analysis).

At each scan, 10 s of observation were performed; data were expressed as the percentage of animals expressing the behavior out of the total number of visible animals at each scan recorded over a 2-h period.

### Statistical methods

Statistical analyses were performed with SPSS version 25 (IBM, SPSS Inc., Chicago, IL) and GraphPad Prism version 7.0 (GraphPad Software, San Diego, CA). The level of statistical significance was set at < 0.05.

Among the evaluated 3 sampling intervals, the best estimate of the true occurrences (i.e., values obtained by continuous sampling method) was chosen on the basis of the R^2^ of regression models, the Mean Absolute Error (MAE), and the Root Mean Square Error (RMSE). Four sessions for each genotype (2 sessions/replicate) were randomly chosen and all the occurrences were determined by the continuous sampling method. These occurrences were then included as the dependent variable in regression models and considered the gold standard for MEA calculation.

The analysis of the behavioral data collected with the 3 sampling intervals aimed to evaluate the following:

i. Reliability, analyzing the agreements among both observers and sampling methods;
ii. Accuracy, calculating error rates and biases;
iii. Validity, verifying the correctness of the inferences that can be made during the practical application of the three sampling intervals.

#### Reliability of the sampling methods: agreement analyses

First, the agreements among observers and among sampling methods were evaluated to determine the reliability of each sampling method^34,35^. The interobserver reliability was estimated with the intraclass correlation coefficient (ICC) using a two-way mixed model for a single measure^35,36^ and using data from individual scans. The ICC values were interpreted as poor (ICC < 0.40), fair (0.40 ≤ ICC < 0.60), good (0.60 ≤ ICC < 0.75), or excellent (ICC ≥ 0.75)^35^. Each ICC was followed by its 95% confidence interval (CI) and by the *p* value of the F test.

Then, the mean proportion of animals engaging in the behavior (out of the total number of animals at each scan recorded over the 2-h period) was calculated for each pen and sampling interval. Data collected by Observer A were used for the subsequent analyses. Moreover, to account for the potential influence of the expected occurrence of a behavior on the reliability of the recording^20^, the behaviors were classified using the binning technique^3^ applied to the recordings at 10-min intervals in the following categories of occurrence:

- Low-occurrence (≤ 0.49% of animals expressing the behavior per scan): Feed pecking, Stretching, Attacking, Fluffing, Escaping, and Allopreening;
- Medium-occurrence (> 0.49% and ≤ 3.50% of animals expressing the behavior per scan): Drinking, Running, Hiding, Dust bathing, Scratching, and Wing flapping;
- High-occurrence (> 3.50% of animals expressing the behavior per scan): Grass pecking, Other pecking, Walking, Resting, Roosting, and Preening.

The variable “Other behaviors” was not included in this categorization.

The agreement among the frequencies obtained with the three sampling methods was evaluated using ICC for average measures adopting the reference ranges described above.

#### Accuracy and validity: differences among sampling methods and their application

The three sampling methods were compared to define their errors and possible differences. Descriptive statistics were used to present the mean proportion of animals per scan engaging each behavior according to the category of occurrence. Related-samples Friedman’s tests were used to investigate whether the proportions of animals engaging in a behavior were influenced by the sampling method. Multiple comparisons were adjusted for with Bonferroni correction. Then, the error scores were calculated^20^ and compared by related-samples Wilcoxon signed-rank tests. The sampling methods were also analyzed using the Bland–Altman approach^38^ to investigate any possible relationship between the measurement error and the best estimate of the reference value (i.e., proportion of animals with the 10-min interval). The Bland–Altman plots were scatter plots in which the y-axis shows the difference between the two measurements (as raw values or percentages) and the x-axis represents their average. The plots also showed the bias (mean difference between measures) and the LoA (± 1.96 standard deviations of the mean difference).

Finally, the influence of the sampling method on the effect of genotype was investigated. A behavioral variable for each category of occurrence was selected (i.e., Walking, Dust bathing, and Attacking) and analyzed for each sampling method with generalized linear models using Tweedie distribution and a log link^39^. The behavioral variables were included as dependent variables, and the genotype was included as an independent variable. Moreover, the day of sampling was included in the models as a within-subject effect and covariate. The effect size of genotype was expressed as the odds ratio (OR) and 95% CI. Estimated effect sizes were compared^40^ using the method described by Altman and Bland^41^ to evaluate whether the observed effect of genotype on each behavior was the same for different sampling methods.

### Supplementary Information

Supplementary Information.

## Data Availability

The datasets used and analyzed during the current study are available from the corresponding author on reasonable request.
