# Supplementary material for: New approaches to selecting a scan-sampling method for chicken behavioral observations and their practical implications
Source: Sci Rep. 2023 Oct 11;13:17177. doi: 10.1038/s41598-023-44126-2 (PMC10567684; doi:10.1038/s41598-023-44126-2)
Supplement: Supplementary file 1 — Supplementary Information. [file 41598_2023_44126_MOESM1_ESM.pdf]

SUPPLEMENTARY INFORMATION

**Behavior**	**SAMPLING INTERVAL 5-min interval**
High-occurrence behaviors	
Roosting	31.70±15.20
Walking	18.24±9.40
Grass pecking	14.60±7.17
Resting	12.39±8.19
Other pecking	6.53±6.68
Self-grooming	3.91±4.02
Medium-occurrence behaviors	
Hiding	3.32±5.59
Running	3.30±4.25
Dust bathing	1.47±3.92
Wing flapping	1.39±1.98
Drinking	0.91±1.68
Scratching	0.54±0.98
Low-occurrence behaviors	
Stretching	0.54±0.98
Attacking	0.45±0.76
Feed pecking	0.08±0.45
Escaping	0.10±0.29
Allo-grooming	0.05±0.16
Swelling	0.06±0.35
Sleeping	0.00±0.03

Supplementary Table S1. Animals performing the different behaviors (% of all visible animals ± standard deviation) using the 5-minutes sampling method.

**Behavior**	**ICC**	**95% CI (lower bound)**	**95% CI (upper bound)**	**P value**
High-occurrence behaviors				
Roosting	0.989	0.982	0.993	***
Walking	0.983	0.970	0.990	***
Grass pecking	0.982	0.970	0.989	***
Resting	0.970	0.946	0.982	***
Other pecking	0.990	0.984	0.994	***
Self-grooming	0.984	0.974	0.990	***
Medium-occurrence behaviors				
Hiding	0.989	0.983	0.994	***
Running	0.976	0.960	0.986	***
Dust bathing	0.993	0.988	0.996	***
Wing flapping	0.979	0.966	0.987	***
Drinking	0.953	0.921	0.972	***
Scratching	0.983	0.972	0.990	***
Low-occurrence behaviors				
Stretching	0.964	0.941	0.978	***
Attacking	0.963	0.919	0.981	***
Feed pecking	0.977	0.963	0.986	***
Escaping	0.938	0.900	0.962	***
Allo-grooming	0.936	0.897	0.961	***
Swelling	0.957	0.930	0.974	***
Sleeping	-	-	-	-

Supplementary Table S2. Agreement among the 5- and 10-minutes sampling intervals methods. Intraclass correlation coefficients (ICCs) of the behavioral variables assessed by Observer A using the 5- and 10-minutes sampling intervals. Each ICC is followed by its 95% confidence interval (CI) and by the P value of the F test. *** P<0.001; - not calculated due to zero variance.

Item	SAMPLING METHOD								
	10-min interval			15-min interval			30-min interval		
	**ICC**	**95%CI**	**P value**	**ICC**	**95%CI**	**P value**	**ICC**	**95%CI**	**P value**
High-occurrence behaviors									
Roosting	0.999	0.998-1.000	***	0.999	0.998-1.000	***	0.998	0.994-0.999	***
Walking	0.998	0.994-0.999	***	0.999	0.998-1.000	***	0.996	0.989-0.999	***
Grass pecking	0.997	0.991-0.999	***	0.997	0.991-0.999	***	0.994	0.983-0.998	***
Resting	0.999	0.997-1.000	***	0.999	0.997-1.000	***	0.996	0.989-0.999	***
Other pecking	0.998	0.995-0.999	***	0.995	0.985-0.998	***	0.991	0.975-0.997	***
Self-grooming	0.999	0.997-1.000	-	0.998	0.995-0.999	-	0.996	0.989-0.999	-
Medium-occurrence behaviors									
Hiding	0.998	0.994-0.999	***	0.996	0.988-0.999	***	0.987	0.964-0.996	***
Running	1.000	0.999-1.000	***	1.000	0.999-1.000	***	1.000	0.999-1.000	***
Dust bathing	1.000	1.000-1.000	***	1.000	0.999-1.000	***	0.999	0.996-1.000	***
Wing flapping	0.999	0.998-1.000	***	0.996	0.990-0.999	***	0.995	0.985-0.998	***
Drinking	0.999	0.998-1.000	***	0.999	0.996-1.000	***	0.999	0.997-1.000	***
Scratching	0.998	0.995-0.999	***	0.998	0.993-0.999	***	0.998	0.995-0.999	***
Low-occurrence behaviors									
Stretching	0.998	0.994-0.999	***	0.998	0.994-0.999	***	0.997	0.992-0.999	***
Attacking	0.991	0.973-0.997	***	0.994	0.984-0.998	***	0.989	0.970-0.996	***
Feed pecking	0.999	0.999-1.000	***	0.998	0.993-0.999	***	0.998	0.993-0.999	***
Escaping	0.994	0.984-0.998	***	0.985	0.957-0.995	***	0.977	0.935-0.992	***
Allo-grooming	0.921	0.790-0.972	***	0.718	0.361-0.892	**	0.715	0.356-0.890	**
Swelling	0.996	0.988-0.999	***	0.993	0.979-0.997	***	0.997	0.992-0.999	***
Sleeping	-	-	-	-	-	-	-	-	-

Supplementary Table S3. Interobserver agreement. Intraclass Correlation Coefficients (ICC) of items included in the ethogram assessed by two observers using the three sampling methods. Each ICC is followed by its 95 % confidence interval (CI) and by the *P*-value of F test. ***P<0.001.

- not calculated due to zero variance

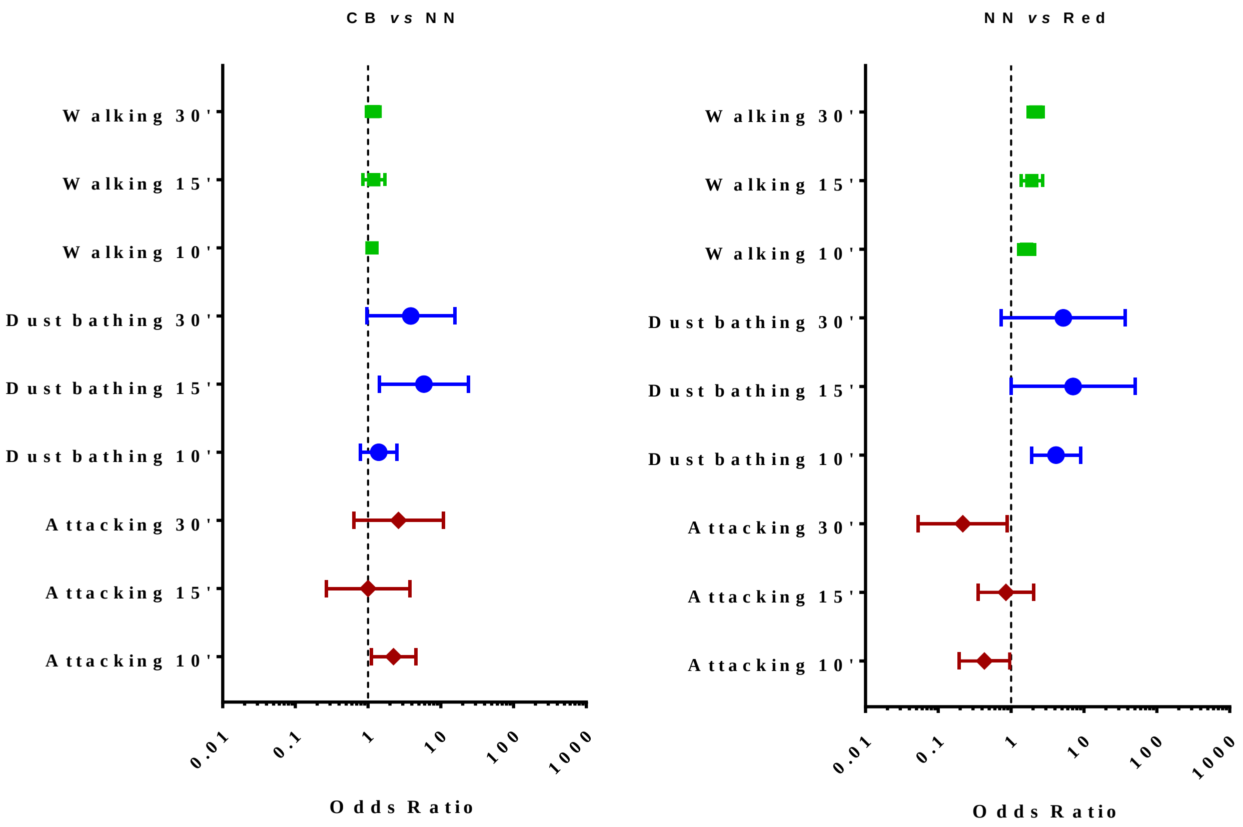

Supplementary Figure S1. Odds ratios for the genotype effect under the three sampling methods on high-, medium- and low-occurrence behaviors (Walking, Dust bathing and Attacking, respectively). Comparisons between these genotypes (i.e., CB *versus* NN and NN *versus* Red) revealed no difference in the odds ratios obtained with the three sampling methods.
